# Supplementary material for: Does Dietary Lipid Level Affect the Quality of Triploid Rainbow Trout and How Should It Be Assessed?
Source: Foods. 2022 Dec 21;12(1):15. doi: 10.3390/foods12010015 (PMC9818296; doi:10.3390/foods12010015)
Supplement: Supplementary file 1 [file foods-12-00015-s001.zip › foods-2041964-supplementary.pdf]

Table S1 Formulation and proximate compositions of the experimental diets (% dry matter)

Ingredients	Diet 1 (6.6%)	Diet 2 (14.8%)	Diet 3 (22.8%)	Diet 4 (29.4%)
Fish meal[1]	60	60	60	60
Wheat meal[1]	12	12	12	12
Corn starch[1]	22.22	14.22	6.22	0.22
Fish oil	0	8	16	22
Soybean oil	3	3	3	3
Mineral premix[2]	0.5	0.5	0.5	0.5
Vitamin premix[3]	0.5	0.5	0.5	0.5
Ca $(H_2PO_4)_2$	0.8	0.8	0.8	0.8
Choline chloride	0.3	0.3	0.3	0.3
Mold inhibitor	0.1	0.1	0.1	0.1
Antioxidants	0.05	0.05	0.05	0.05
Betaine	0.5	0.5	0.5	0.5
Astaxanthin[4]	0.03	0.03	0.03	0.03
Total	100	100	100	100
Proximate analysis (n=3)				
Moisture (%)	4.3	3.7	4.3	4.2
Crude protein (% dry matter)	46.3	46.1	45.8	45.5
Crude lipid (% dry matter)	6.6	14.8	22.8	29.4
Ash (% dry matter)	11.1	11.1	10.9	10.9
Gross energy (KJ /g)	18.9	21.1	22.9	24.2

[1] Fish meal, crude protein 69.5%, crude lipid 8%. Wheat meal, crude protein 15%, crude lipid 1.2%. Corn starch, crude protein 0.3%, crude lipid 0.2%.

[2] Mineral premix included the following (mg /kg diet): sodium, 1500; iron, 3000; copper, 90; zinc, 1500; manganese, 800; selenium, 4.3; iodine, 21; cobalt, 3.

[3] Vitamin premix included the following (each kg^{-1} diet): vitamin A, 50 KIU; vitamin D3, 20 KIU; vitamin E, 390 mg; vitamin K, 150 mg; vitamin B1, 120 mg; vitamin B2, 165 mg; vitamin B6, 130 mg; vitamin B12, 0.5 mg; biotin, 2.4 mg; folic acid, 75 mg; inositol, 1200 mg; niacin, 670 mg; ascorbic acid, 2500 mg.

[4] Astaxanthin: 10% (CAROPHYLL®, DSM, Netherlands).
